# Notified cases of mpox in the city of Rio de Janeiro, Brazil: a descriptive study, 2022

**DOI:** 10.1590/S2237-96222024v33e2023899.en

**Published:** 2024-04-15

**Authors:** Caio Luiz Pereira Ribeiro, Camila Arantes Ferreira Brecht D’Oliveira, Élida de Albuquerque Campos, Luciana Freire de Carvalho, Luciana de Almeida Pinto, Karoline Moreira Duffrayer, Poliana Hilário Magalhães, Raquel Proença, José Cerbino, Gislani Mateus Oliveira Aguilar, Márcio Henrique de Oliveira Garcia

**Affiliations:** 1 Secretaria de Vigilância em Saúde do Rio de Janeiro, Coordenação de Informações Estratégicas de Vigilância em Saúde, Rio de Janeiro, RJ, Brazil; 2 Ministério da Saúde, Departamento de Análise Epidemiológica e Vigilância de Doenças Não Transmissíveis, Brasília, DF, Brazil; 3 Secretaria de Vigilância em Saúde do Rio de Janeiro, Superintendência de Vigilância em Saúde do Rio de Janeiro, Rio de Janeiro, RJ, Brazil; 4 Ministério da Saúde, Departamento de Emergências em Saúde Pública, Brasília, DF, Brazil; 5 Fundação Oswaldo Cruz, Rio de Janeiro, RJ, Brazil

**Keywords:** Monkeypox, Outbreaks, Epidemiology, Public Health Surveillance, Public Health, Monkeypox, Brotes, Epidemiología, Vigilancia en Salud Pública, Salud Pública, Monkeypox, Surto, Epidemiologia, Vigilância em Saúde Pública, Saúde Pública

## Abstract

**Objective:**

To describe the profile of cases of mpox in the city of Rio de Janeiro between June and November 2022.

**Methods:**

This was a descriptive study of secondary data obtained from mpox notification forms. Socioeconomic, clinical and spatial data were analyzed.

**Results:**

Of the 928 cases, 93.7% were male, 85.0% cisgender male, 65.6% homosexual, 41.8% between 30 and 39 years old, and 41.0% were of White race/skin color. A total of 34.5% had immunosuppression due to illness, and 41.9% reported their HIV status as being positive. The most prevalent signs and symptoms were: skin lesions (96.6%), especially with multiple manifestations (67.8%) in the genital region (46.1%), in addition to fever (58.3%), adenomegaly (43.3%) and headache (38.7%). Most notifications occurred in public services (81.3%) and in hospital care (51.3%).

**Conclusion:**

The study revealed high incidence of mpox, especially among young, cisgender and homosexual men. Most cases were mild, with genital lesions, progressing to cure without hospitalization. Person-to-person transmission was predominant.

## Study contributions

**Main results:**

The notified cases of mpox in the city of Rio de Janeiro were mainly concentrated in men aged 30 to 39 years. The majority presented a mild condition that progressed to cure without hospitalization.

**Implications for services:**

The profile obtained can contribute to the targeting of local health care policies, targeting prevention and health promotion actions.

**Perspectives:**

Additional investigations can contribute to expanding knowledge of the disease. Investment in health surveillance is necessary to respond to public health emergencies.

## RESUMEN

**Objetivo:**

Describir el perfil de los casos de mpox en la ciudad de Río de Janeiro entre junio y noviembre de 2022.

**Métodos:**

Estudio descriptivo de datos secundarios de los formularios de notificación de mpox. Se analizaron datos socioeconómicos, clínicos y espaciales.

**Resultados:**

De los 928 casos, 93,7% eran hombres, 65,6% homosexuales, 41% blanca, y 41,8% de 30 a 39 años. Estaban inmunocomprometidos debido a una enfermedad 34,5%, y 41,9% reportaron serología positiva para virus de inmunodeficiencia humana. Signos y síntomas más prevalentes fueron: lesiones cutáneas (96,6%), con manifestaciones múltiples (67,8%) y en la región genital (46,1%), además de fiebre (58,3%), adenomegalia (43,3%) y cefalea (38,7%). Mayoría de las notificaciones ocurrieron en la red pública (81,3%) y atención hospitalaria (51,3%).

**Conclusión:**

El estudio reveló alta incidencia de mpox, especialmente entre hombres jóvenes, cisgénero y homosexuales. Mayoría de los casos tuvieron una presentación leve, progresando hasta la curación sin hospitalización.

## RESUMO

**Objetivo:**

Descrever o perfil dos casos de mpox na cidade do Rio de Janeiro, entre junho e novembro de 2022.

**Métodos:**

Estudo descritivo de dados secundários das fichas de notificação de mpox. Foram analisados dados socioeconômicos, clínicos e espaciais.

**Resultados:**

Dos 928 casos, 93,7% foram do sexo masculino, 85,0% homem cisgênero, 65,6% homossexual, 41,8% entre 30 e 39 anos e 41,0% brancos. Apresentavam imunodepressão por doença 34,5%, e 41,9% informaram sorologia positiva prévia para vírus da imunodeficiência humana. Os sinais e sintomas mais prevalentes foram: lesões cutâneas (96,6%), especialmente com manifestações múltiplas (67,8%) e em região genital (46,1%), além de febre (58,3%), adenomegalia (43,3%) e cefaleia (38,7%). A maioria das notificações ocorreu na rede pública (81,3%) e na atenção hospitalar (51,3%).

**Conclusão:**

O estudo revelou maior incidência de mpox entre homens cisgênero, autodeclarados de cor branca e homossexual. A maioria dos casos teve apresentação leve evoluindo para cura sem hospitalização.

## INTRODUCTION

Mpox is a zoonotic viral disease with an average incubation period of 6 to 16 days, the reservoir of which is unknown.^1^ The first cases were associated with hunting and eating infected wild animals,^2^ although its transmission also occurs between people or through contact with infected material.^3,4^ The most common signs and symptoms of the disease are skin lesions, headache, fever, myalgia, fatigue and lymph node enlargement,^5^ and transmission ends once the lesions have disappeared. Most cases have mild clinical manifestations and have good prognosis, however, in immunosuppressed individuals, the condition may worsen and require hospitalization.^4^

Mpox is an abbreviation for monkeypox, as it was identified in a non-human primate in 1958.^3^ In 1970, the first case in humans was identified in the Democratic Republic of the Congo,^2^ with outbreaks occurring in the 1970s and 1980s in that republic and in other countries in the West and Central Africa.^3^ In recent years, there has been an increase in the incidence rate from 0.72 cases per 100,000 inhabitants, in 1980, to 14.4 cases/100,000 inhab. in 2000, on the African continent.^3^ In 2003, the United States had its first outbreak of mpoxs^6^ and, in 2017 and 2018, there were outbreaks in Nigeria and Cameroon, where there had been no recorded cases for two decades.^3^ From May to July 2022, more than 16,000 cases of mpox were reported in 75 countries,^7^ with the World Health Organization (WHO) declaring a Public Health Emergency of International Concern in relation to the mpox outbreak.^8^ In Brazil, 10,904 confirmed mpox notifications had been registered by Epidemiological Week (EW) 16 of 2023,^9^ of which 1,039 (9.5%) notifications related to confirmed cases in the city of Rio de Janeiro.^10^

The scientific literature on mpox is still scarce.^11^ In the same way as other endemic diseases on the African and South American continents, this health problem has been neglected, gaining repercussion and research funding only after the occurrence of cases in the Northern Hemisphere, revealing the colonial perspective in scientific practice.^12^ The importance of understanding the manifestation of the disease in the population is highlighted, so as to contribute to building health actions. As such, the objective of this article was to describe the sociodemographic and clinical characteristics of reported mpox cases among residents of the city of Rio de Janeiro.

## METHODS

### Design and data source

This was a descriptive study of mpox data reported to the Ministry of Health. The Research Electronic Data Capture (REDCap) tool was used to manage the database, which was structured, validated and managed by the Rio de Janeiro Municipal Health Department. 

### Study participants

The study included confirmed mpox cases among people resident in the city of Rio de Janeiro, Brazil, registered on the aforementioned platform from June 15, 2022 – date of the first mpox case notified in the city of Rio de Janeiro – until November 7, 2022, the closing date for notifications input to REDCap. 

### Context

This study used case definitions according to risk communications about mpox issued by the Rio de Janeiro Strategic Health Surveillance Information Coordination Service (*Coordenação de Informação Estratégica em Vigilância em Saúde*) in the city of Rio de Janeiro, adapted from Ministry of Health definitions (Supplementary Box 1). A suspect case is considered to be an individual of any age who, with effect from March 15 2022, has sudden onset of acute skin rash suggestive of mpox, either single or multiple, on any part of their body (including the genital region), whether or not associated with adenomegaly or report of fever. A confirmed mpox case is an individual who meets the suspect case definition and has a “positive/detectable” mpox laboratory test result/report obtained by means of molecular diagnosis (real-time PCR and/or sequencing).

### Variables

The variables analyzed were sex (“male”, “female”); age in years and age group (“0-9”, “10-19”, “20-29”, “30-39”, “40-49”, “50-59”, “60+”); gender identity (“cisgender man”, “cisgender woman”, “transgender man”, “transgender woman”, “non-binary” and “not informed”); sexual orientation (“homosexual”, “heterosexual”, “bisexual”, “pansexual”, “other” and “not informed”); race/skin color (“White”, “mixed race”, “Black”, “Asian”, “Indigenous” and “not informed”); schooling (“illiterate”, “incomplete elementary education”, “complete elementary education”, “complete high school education”, “complete higher education”, “not applicable” and “not informed”); nationality (open field); immunosuppression (“due to illness”, “due to medication”, “cause unknown”, “not immunosuppressed” and “not informed”); HIV positive (“yes”, “no” and “not informed”); active sexually transmitted infection (“yes”, “no” and “not informed”); type of active sexually transmitted infection (“chlamydia”, “gonorrhea”, “genital herpes”, “*lymphogranuloma venereum*”, “*mycoplasma genitalium*” “syphilis”, “trichomoniasis”, “genital warts”, “chancroid” “human papillomavirus”, “pelvic inflammatory disease”, “donovanosis”, “human T-lymphotropic virus infection”, “other”); signs and symptoms (open field); site of lesion (“face”, “torso”, “lower limbs”, “upper limbs”, “genital”, “anal”, “oral”, “palm of the hand”, “sole of the feet”, “other places”); probable form of transmission (“from animal to man”, “associated with health care”, “transmitted in a laboratory”, “due to exposure at work”, “contact with contaminated material”, “person-to-person”, “transmission via use of intravenous drugs and blood transfusion”, “vertical transmission”, “sexual transmission”, “other transmission”, “unknown”, “not informed”); hospitalization (“yes ‒ due to clinical needs”, “yes ‒ for isolation”, “no”, “not informed”); progression (“unknown”, “cure”, “death from mpox”, “death from other cause”); place of residence in the city of Rio de Janeiro (open field); notifying service (open field); notifying health system (“public”, “private”, “military”); date of symptom onset (dd/mm/yyyy).

**Figure 1:**
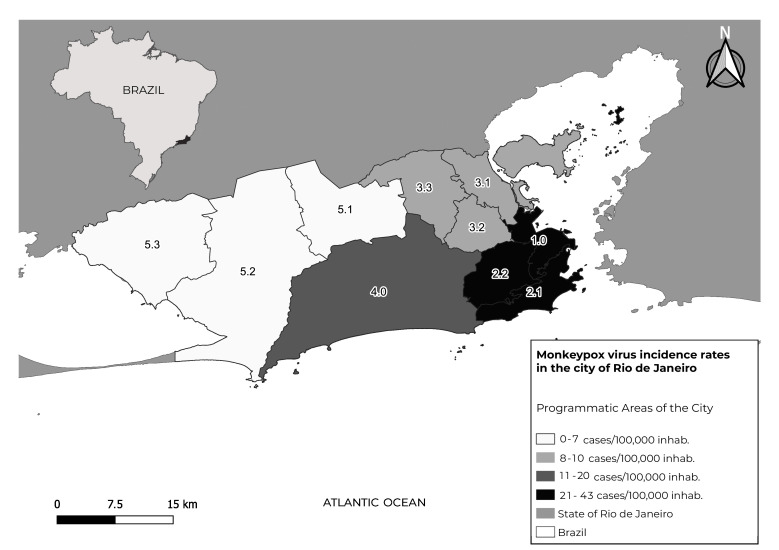
Distribution of mpox rates per programmatic area of residence of cases in the city of Rio de Janeiro, from June to November 2022 (n = 928)

Programmatic areas were taken as the unit of analysis regarding place of residence. The city of Rio de Janeiro is divided into ten health programmatic areas, which are organized according to the city’s administrative regions, which, in turn, are comprised of neighborhoods. The ten programmatic areas are distributed in the Central region (programmatic area 1.0), South Zone (programmatic area 2.1), North Zone (programmatic areas 3.1, 3.2 and 3.3) and West Zone (programmatic areas 4.0, 5.1, 5.2 and 5.3) of the city (Figure 1).

The estimated population of the city of Rio de Janeiro was obtained from the population projection for the year 2022 made available by the *Instituto Pereira Passos*.^13^ The populations of the administrative regions were grouped into the ten programmatic areas of the city of Rio de Janeiro, and were then used as a denominator to calculate the mpox incidence rates for each territory. After calculation, these data were plotted in order to visualize the spatial distribution of mpox rates in the city of Rio de Janeiro in the period analyzed.

### Data analysis 

Descriptive statistical analyses of the demographic, socioeconomic and clinical data of confirmed cases of mpox were performed out using R software version 4.2.1, and measures of central tendency and dispersion of continuous variables and frequencies of categorical variables were calculated. 

QGIS software version 3.18.1 “Zürich” was used to describe the spatial distribution of incidence rates, whereby analyses were performed according to the programmatic areas, through shapefiles made available by the *Instituto Brasileiro de Geografia e Estatística* 14 and by the *Instituto Pereira Passos*.^15^

### Data quality control 

Database records underwent quality control to eliminate any duplicate records or to modify records with inconsistent data.

### Ethical aspects 

This study was approved by the Rio de Janeiro Municipal Health Department Research Ethics Committee, as per Opinion No. 5.739.997 on 04/11/2022, Certificate of Submission for Ethical Appraisal (*Certificado de Apresentação de Apreciação Ética*) No. 64021122.6.0000.5279.

## RESULTS

**Figure 2:**
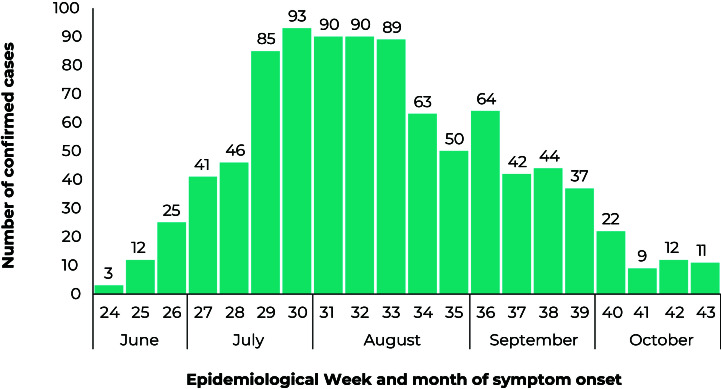
Distribution of mpox virus cases in the city of Rio de Janeiro by epidemiological week of onset of symptoms, from June to November 2022 (n = 928)

All 928 confirmed cases of mpox resident in the city of Rio de Janeiro notified during the study period were included. The onset of symptoms of the first case occurred in EW 24 of 2022, and the peak in cases can be seen in EW 30 of 2022 (Figure 2).

The cases were predominantly of the male sex (93.7%), between 20 and 49 years old (91.3%) ‒ with a median age of 34 years (range: 0-93 years), cisgender (91.1%), homosexual (65.6%), of White race/skin color (41.0%) and had complete high school education (44.2%) (Table 1). 

**Table 1:** Confirmed mpox cases resident in the city of Rio de Janeiro, according to sociodemographic characteristics, from June to November 2022 (n = 928)

Characteristics	N	%
**Sex**		
Male	870	93.7
Female	58	6.3
**Age group (years)**		
0-9	7	0.8
10-19	20	2.2
20-29	261	28.1
30-39	388	41.8
40-49	199	21.4
50-59	45	4.8
60+	8	0.9
**Gender identity**		
Cisgender man	789	85.0
Cisgender woman	57	6.1
Transgender woman	12	1.3
Non-binary	7	0.8
Transgender man	2	0.2
Not informed	61	6.6
**Sexual orientation**		
Homosexual	609	65.6
Heterosexual	144	15.5
Bisexual	99	10.7
Pansexual	20	2.1
Other	7	0.8
Not informed	49	5.3
**Race/skin color**		
White	380	41.0
Mixed race	333	35.9
Black	161	17.3
Asian	24	2.6
Indigenous	2	0.2
Not informed	28	3.0
**Schooling**		
Illiterate	1	0.1
Incomplete elementary education	32	3.5
Complete elementary education	54	5.8
Complete high school education	410	44.2
Complete higher education	337	36.3
Not applicable	5	0.5
Not informed	89	9.6

There were no pregnant women in the sample and the majority of cases (98.8%) were of Brazilian nationality. Of the 11 foreigners, 5 were Argentinian, 2 were Colombian, 2 were French, 1 was from Bolivia and 1 was from Peru.

Among the notified cases, 34.5% had immunosuppression due to diseases, 41.9% reported being HIV positive, 13.2% reported having some other active sexually transmitted infection at the time of care, and of these, 85.2% had active syphilis at the time of mpox notification (laboratory confirmed) (Table 2). The most common mpox signs and symptoms were: lesion in genital/perianal/oral skin tissue/mucous membrane (96.6%), fever (58.3%), adenomegaly (43.3%), headache (38.7%). Lesions predominated in multiple sites on the body (67.8%), the most common being in the genital region (46.1%). The main form of transmission, assumed based on the information contained in the notification, was person-to-person (33.0%), followed by sexual transmission (19.2%). No hospitalization occurred in the majority of cases (94.7%) and the majority of individuals recovered (89.3%) (Table 2). Other signs and symptoms were reported less frequently, which characterized atypical conditions of the disease (Supplementary Table 1).

**Table 2:** Confirmed mpox cases resident in the city of Rio de Janeiro, according to self-reported clinical manifestations, from June to November 2022 (n = 928)

Characteristics	N	%
Immunosuppression		
Comorbidity	320	34.5
Due to medication	6	0.7
Cause unknown	2	0.2
Not immunosuppressed	505	54.4
Not informed	95	10.2
**HIV positive**		
Yes	389	41.9
No	486	52.4
Not informed	53	5.7
**Active sexually transmitted infection**		
Yes	122	13.2
No	645	69.5
Not informed	161	17.3
**Types of active sexually transmitted infection**		
Syphilis	104	85.3
Gonorrhea	5	4.1
Chlamydia	2	1.6
Genital herpes	2	1.6
Other	9	7.4
**Signs and symptoms** ^a^		
Skin/mucous membrane/perianal/oral lesion	896	96.6
Fever	541	58.3
Adenomegaly	403	43.4
Headache	359	38.7
Asthenia/weakness	286	30.8
Muscle pain	268	28.9
Sore throat	222	23.9
Sweating/shivering	207	22.3
Backache	120	12.9
Other	799	86.0
**Characteristic of the lesion**		
Single	72	7.8
Multiple	629	67.8
Not informed	227	24.4
**Site of the lesion**		
Genital	428	46.1
Upper limbs and palms	334	36.0
Face	294	31.7
Torso	289	31.1
Lower limbs and soles of feet	227	24.5
Anal	82	8.8
Oral	58	6.3
Other sites	20	2.2
Not informed	225	24.2
**Probable form of transmission**		
Person-to-person^b^	306	33.0
Sexual transmission	178	19.2
Associated with health care	3	0.3
Contact with contaminated material	2	0.2
Unknown	191	20.6
Not informed	248	26.7
**Hospitalization**		
Due to clinical needs	42	4.6
For isolation	4	0.4
Not hospitalized	879	94.7
Not informed	3	0.3
**Case progression**		
Cure	829	89.3
Death from other cause	1	0.1
Not informed	98	10.6

Regarding the location of case notification, 51.3% occurred in hospitals, 39.9% in Primary Health Care Centers, whereby 81.3% of total care was provided in public health system services (Table 3). Regarding place of residence, all ten programmatic areas in the city of Rio de Janeiro had resident cases registered, with the highest concentration of cases in programmatic area 2.1 (25.8%), followed by programmatic area 4.0 (17.2) and programmatic area 1.0 (14.0%) (Table 3). 

**Table 3:** Confirmed mpox cases resident in the city of Rio de Janeiro, according to programmatic area of residence and notifying service characteristics, from June to November 2022 (n = 928)

Characteristics	N	%
**Programmatic area of residence**		
1.0	130	14.0
2.1	239	25.8
2.2	84	9.0
3.1	86	9.3
3.2	60	6.5
3.3	72	7.8
4.0	160	17.2
5.1	46	5.0
5.2	27	2.9
5.3	24	2.5
**Notifying service**		
Hospital	476	51.3
Primary care	370	39.9
Emergency Care Unit or Regional Emergency Coordination	41	4.4
Others	41	4.4
**Notifying health system**		
Public	754	81.3
Private	162	17.4
Military	12	1.3

Regarding the incidence rate, the highest rate was found in programmatic area 1.0, with 43 cases per 100,000 inhab., followed by programmatic area 2.1 and programmatic area 2.2, with 39 and 21 cases per 100,000 inhab., respectively (Figure 1).

## DISCUSSION

This study described the profile of confirmed cases of mpox living in the city of Rio de Janeiro, which occurred, for the most part, among young male adults, with a cisgender identity and homosexual sexual orientation, in addition to people who reported being of White race/skin color and having completed high school education.

This study has some limitations. During this analysis period, the notification form was updated three times, resulting in changes and updates to variables, including new mandatory fields. This generated gaps in the initial forms. Furthermore the difficulty of migrating and integrating data, together with changes to the notification form, led to some information being incomplete. 

The epidemiological profile was similar to that described in European countries, which also showed higher incidence of cases in the population of young male adults, with homosexual and bisexual sexual orientation and among men who have sex with men.^17-20^ It is essential to highlight that the disease is not exclusive to a certain gender or sexual orientation, and this reflection must be emphasized to combat any stigma that may be associated with the population most at risk. When comparing the results of this research with data for Brazil as a whole,^21^ it is possible to corroborate the trends found, with a peak in cases between EWs 30-31, followed by a drop in the occurrence of the disease. The epidemiological and clinical profile observed in the city of Rio de Janeiro was also similar to the national one, with higher incidence in cisgender men, of White race/skin color, in the 30 years age group, presenting mild symptoms and with good case progression.^21^

Several findings of this study are in line with the clinical characteristics described by the WHO.^2-23^ It is important to highlight that although the occurrence of multiple lesions is the most frequent clinical characteristic,^17^ many people infected by the virus may be asymptomatic.^24^ Although the cases were predominantly mild, it is important to highlight the high prevalence of risk factors for severity.

We found a high percentage of people with positive HIV serology, and mpox and HIV co-infection could explain a possible greater severity of clinical manifestations, although this was not found in our sample.^25^ It is possible that the severity of the cases was low due to the timeliness of case diagnosis and treatment.

We found higher incidence of cases in central areas with greater purchasing power in the city of Rio de Janeiro (programmatic areas 1.0 and 2.1). These areas are characterized by the presence of leisure facilities and concert halls, in addition to being the territories most visited by tourists who come to the city of Rio de Janeiro.^26^ These factors may provide more opportunities for prolonged contact between individuals, as well as increasing the likelihood of interaction with people from other countries or regions where the virus is circulating. 

Public hospitals and health centers made most case notifications and, more than a reflection of the severity or socioeconomic profile of the cases, this distribution may be linked to the referral strategy adopted by the public health authorities in the city of Rio de Janeiro. As the mpox outbreak was a Public Health Emergency of International Concern, the city ​​concentrated attention on the first cases at a reference unit for infectious disease clinical research, teaching, referral services and care, this being a fact that facilitated the care and surveillance services to achieve timely epidemiological investigation and case monitoring.

Around 1% of mpox cases in the world were residents of the city of Rio de Janeiro,^5^ which accounted for 74% of cases in the state of Rio de Janeiro^16^ and 9% of cases in Brazil.^9^ Although there were no deaths among residents of the city of Rio de Janeiro, Brazil was the country with the second highest absolute number of deaths^5^ and the state of Rio de Janeiro had the highest number of deaths in Brazil.^9^

Considering the epidemiological panorama of mpox infection in Brazil,^9^ despite the decreasing trend in the world,^5^ the United States Centers for Disease Control and Prevention stressed the importance of monitoring and preventive interventions among people living with HIV due to the serious manifestations in this population group. This guidance was given in view of the frequency of deaths and the greater occurrence of morbidity and mortality among people living with HIV, specifically those with CD4+ T lymphocyte counts < 200 cells.^27^ As such, in March 2023, the Ministry of Health began its pre-exposure vaccination campaign in Brazil against mpox aimed at this population using the immunizing agent MVA-BN Jynneos mpox.^3,5^ At an individual level, vaccination should not replace other known protective measures.^27^

This study is the first to analyze the profile of mpox cases in the city of Rio de Janeiro, contributing to a better understanding of this problem in the population and providing support for effective health actions. Case management also reflects the central and fundamental role of Health Surveillance in the city of Rio de Janeiro, with emphasis on the Rapid Response Unit teams of the Strategic Health Surveillance Information Coordination Service, which allowed detection and timely notification of cases, enabling the implementation of control actions, such as contact tracing, isolation and monitoring of individuals until the cases were closed. This study also helps to highlight the importance of investing in Health Surveillance as an essential tool for responding to public health emergencies, fundamental in advancing the field of public health, as well as highlighting the need for additional investigations and studies to continue tackling this health problem.

In conclusion, this study offered a comprehensive analysis of the profile of mpox cases in the city of Rio de Janeiro, in 2022. The disease predominantly affected young male adults, self-declared homosexuals and of White race/skin color. In most cases, transmission occurred directly, presenting mild symptoms and progressing to cure without hospitalization. 

## Supplementary Table 1

**Table:** Other signs and symptoms of confirmed mpox cases resident in the city of Rio de Janeiro, from June to November 2022 (n = 928)

Signs and Symptoms^a^	N	**%**
Nausea/vomiting	79	8.5
Arthralgia	65	7.0
Localized lymphadenopathy	64	6.9
Pruritus	56	6.1
Pain in other places	55	6.0
Cough	54	5.8
Difficulty swallowing/odynophagia	48	5.2
Diarrhea	43	4.6
Other flu-like symptoms (hoarseness, sneezing, runny nose, congested nose)	41	4.4
Photosensitivity	36	3.9
Fatigue/tiredness/exhaustion	31	3.3
Proctitis	30	3.2
Edema (penis/anus)	29	3.1
Urinary incontinence	22	2.4
Signs of hemorrhaging	21	2.3
Dysuria/burning when urinating/urinary retention	17	1.8
Anal/rectal/vaginal secretion and/or discharge	17	1.8
Hyperemia/exudation	15	1.6
Conjunctivitis	14	1.5
Generalized lymphadenopathy	11	1.2
Malaise	5	0.5
Dyspnea/chest pain/tight ribs	4	0.4
Exanthema	4	0.4
Anal bleeding	4	0.4
Confusion	3	0.3
Hematuria	3	0.3
Irritability	3	0.3
Ulcer	3	0.3
Anal fissure	3	0.3
Eye burning	3	0.3
Lack of appetite/hyporexia	3	0.3
Dizziness	2	0.2
Anorexia	1	0.1
Ageusia	1	0.1
Gastrointestinal alterations	1	0.1
Balanitis	1	0.1
Dry mouth	1	0.1
Shivering	1	0.1
Lumbosacralgia	1	0.1
Paraphimosis	1	0.1
Blushing	1	0.1
Night sweats	1	0.1
Tachypnea	1	0.1

a) An individual could report more than one sign/symptom occurring in the 21 days prior to the medical consultation.

## Supplementary Box 1

**Table:** Description of the guidance documents relating to the definition of decisions on Mpox in the city of Rio de Janeiro, 2022

**Version 01** Issued on 07/06/2022	**Suspect case definition:** Individual of any age who, with effect from March 15 2022, has sudden onset of fever, adenomegaly and acute skin rash of the papulovesicular type and progressing uniformly. **Probable case definition:** Individual who meets the suspect case definition AND one OR more of the following criteria: Have an epidemiological link (close and prolonged exposure without respiratory protection; direct physical contact, including sexual contact; or contact with contaminated materials, such as clothes and bedclothes) with a probable or confirmed monkeypox case, since March 15 2022, in the 21 days prior to the onset of signs and symptoms OR History of traveling to a country that is endemic or had confirmed monkeypox cases in the 21 days prior to the onset of symptoms AND without laboratory confirmation. **Confirmed case definition:** Individual who meets the suspect case or probable case definition with laboratory confirmation of having the monkeypox virus obtained by means of molecular testing (qPCR and/or sequencing). **Discarded case definition:** Suspected case that does not meet the monkeypox confirmation criterion or who had another disease confirmed by means of clinical or laboratory diagnosis.
**Version 02** Issued on 17/06/2022	**Suspect case definition:** Individual of any age who, with effect from March 15 2022, has sudden onset of acute skin rash suggestive of monkeypox, either single or multiple, on any part of their body (including the genital region), whether or not associated with adenomegaly or report of fever AND History of traveling to a country that is endemic or had confirmed monkeypox cases in the 21 days prior to the onset of symptoms OR Have an epidemiological link with people with a history of traveling to a country that is endemic or had confirmed monkeypox cases, since March 15 2022, in the 21 days prior to the onset of signs and symptoms OR Have an epidemiological link with suspected, probable or confirmed monkeypox cases, since March 15 2022, in the 21 days prior to the onset of signs and symptoms OR History of intimate contact with strangers and/or casual partners, in the 21 days prior to the onset of signs and symptoms. **Probable case definition:** Suspect case, submitted to clinical and epidemiological investigation, AND who developed a clinical condition compatible with monkeypox, but without possibility of laboratory confirmation by means of qPCR and/or sequencing. **Confirmed case definition:** Individual who meets the suspect case definition and has a “positive/detectable” monkeypox laboratory test result/report obtained by means of molecular diagnosis (real-time PCR and/or sequencing). **Discarded case definition:** Individual who meets the suspect case definition but has a “negative/undetectable” monkeypox laboratory test result/report obtained by means of molecular diagnosis (real-time PCR and/or sequencing).
**Version 03** Issued on 29/06/2022	**Suspect case definition:** Individual of any age who, with effect from March 15 2022, has sudden onset of acute skin rash suggestive of monkeypox, either single or multiple, on any part of the body (including the genital region), whether or not associated with adenomegaly or report of fever AND History of intimate contact with strangers and/or casual partners, in the 21 days prior to the onset of signs and symptoms OR Have an epidemiological link with confirmed monkeypox cases, since March 15 2022, in the 21 days prior to the onset of signs and symptoms OR History of traveling to a country that is endemic or had confirmed monkeypox cases in the 21 days prior to the onset of symptoms OR Have an epidemiological link with people with a history of traveling to a country that is endemic or had confirmed monkeypox cases, since March 15 2022, in the 21 days prior to the onset of signs and symptoms. **Probable case definition:** Suspect case, submitted to clinical and epidemiological investigation, AND who developed a clinical condition compatible with monkeypox, but without possibility of laboratory confirmation by means of qPCR and/or sequencing. **Confirmed case definition:** Individual who meets the suspect case definition and has a “positive/detectable” monkeypox laboratory test result/report obtained by means of molecular diagnosis (real-time PCR and/or sequencing). **Discarded case definition:** Individual who meets the suspect case definition but has a “negative/undetectable” monkeypox laboratory test result/report obtained by means of molecular diagnosis (real-time PCR and/or sequencing), OR Suspect case who during clinical, epidemiological and laboratory investigation was diagnosed as having another disease compatible with the clinical condition the patient presented, except STI.
**Version 04** Issued on 27/07/2022	**Suspect case definition:** Individual of any age who, with effect from March 15 2022, has sudden onset of acute skin rash suggestive of monkeypox, either single or multiple, on any part of their body (including the genital region), whether or not associated with adenomegaly or report of fever. **Confirmed case definition:** Individual who meets the suspect case definition and has a “positive/detectable” monkeypox laboratory test result/report obtained by means of molecular diagnosis (real-time PCR and/or sequencing). **Probable case definition:** Suspect case, submitted to clinical and epidemiological investigation, AND who developed a clinical condition compatible with monkeypox, but without possibility of laboratory confirmation by means of qPCR and/or sequencing. **Discarded case definition:** Individual who meets the suspect case definition but has a “negative/undetectable” monkeypox laboratory test result/report obtained by means of molecular diagnosis (real-time PCR and/or sequencing), OR Suspect case who during clinical, epidemiological and laboratory investigation was diagnosed as having another disease compatible with the clinical condition the patient presented, except STI.

Source: *Comunicação de Risco, Coordenação de Informação Estratégica de Vigilância em Saúde* (CIEVS Rio).
